# DNA methylation signatures for chromatinopathies: current challenges and future applications

**DOI:** 10.1007/s00439-023-02544-2

**Published:** 2023-04-06

**Authors:** Zain Awamleh, Sarah Goodman, Sanaa Choufani, Rosanna Weksberg

**Affiliations:** 1 Genetics and Genome Biology Program, Research Institute, The Hospital for Sick Children, Toronto, ON Canada; 2 Department of Paediatrics, Division of Clinical and Metabolic Genetics, The Hospital for Sick Children, 555 University Ave, Toronto, ON M5G 1X8 Canada; 3 Department of Molecular Genetics, University of Toronto, Toronto, ON Canada; 4 Institute of Medical Sciences, University of Toronto, Toronto, ON Canada

## Abstract

Pathogenic variants in genes that encode epigenetic regulators are the cause for more than 100 rare neurodevelopmental syndromes also termed “chromatinopathies”. DNA methylation signatures, syndrome-specific patterns of DNA methylation alterations, serve as both a research avenue for elucidating disease pathophysiology and a clinical diagnostic tool. The latter is well established, especially for the classification of variants of uncertain significance (VUS). In this perspective, we describe the seminal DNA methylation signature research in chromatinopathies; the complex relationships between genotype, phenotype and DNA methylation, and the future applications of DNA methylation signatures.

## Introduction

There are more than 100 Mendelian neurodevelopmental syndromes known to be caused by pathogenic variants in epigenetic regulatory genes. Many of these genes encode chromatin modifiers, giving rise to terms such as ‘chromatinopathies’ and ‘Mendelian disorders of epigenetic machinery (MDEM)’ (Fahrner and Bjornsson 2019). These syndromes, often characterized by intellectual disability, growth dysregulation, and congenital anomalies, highlight the link between epigenetic regulation and neurodevelopment. Genome-wide dysregulation of DNA methylation associated with these neurodevelopmental syndromes has been well documented over the past decade, especially for disorders caused by pathogenic variants in genes encoding histone modifiers (Fahrner and Bjornsson 2019). Syndrome-specific patterns of DNA methylation alterations, termed “DNA methylation signatures”, have established clinical diagnostic utility, especially for the classification of variants of uncertain significance (VUS) (Aref-Eshghi et al. 2018; Butcher et al. 2017; Choufani et al. 2015, 2020; Levy et al. 2022). To date, there are ~ 70 DNA methylation signatures that have been developed and a subset of these are currently in use at diagnostic laboratories for patient variant classification in the United States and Europe (Levy et al. 2022). We have demonstrated the utility of DNA methylation signatures beyond diagnostics, in the elucidation and interpretation of complex associations between genotype and phenotype. We are now moving into a new era of DNA methylation signature application. In this perspective, we aim to (i) provide an overview of the early discovery of DNA methylation signatures in neurodevelopmental syndromes; (ii) highlight complex relationships between genotype, phenotype, and DNA methylation “epigenotype”; and iii) discuss the future potential applications of DNA methylation signatures based on the knowledge and insights gained from the first decade of signature research.

## Origins of DNA methylation signatures

In 2013, our group was the first to identify DNA methylation changes in peripheral blood associated with the Intellectual developmental disorder, X-linked syndromic, Claes-Jensen type [MIM:300534] caused by pathogenic variants in the epigenetic regulatory gene *KDM5C* [MIM:314690] (Lysine Demethylase 5C (Grafodatskaya et al. 2013). We used the term DNA methylation “signature” to describe a set of DNA methylation alterations identified in a cohort of individuals with pathogenic variants in a gene known to cause a specific neurodevelopmental syndrome (as compared to healthy “controls”). In 2015, we identified a DNA methylation signature for Sotos syndrome (MIM: 117,550) caused by pathogenic variants in the epigenetic regulatory gene, *NSD1* [MIM:606681] (Nuclear Receptor Binding SET Domain Protein 1) (Choufani et al. 2015). We also demonstrated for the first time the diagnostic utility of DNA methylation signatures in classifying VUS in *NSD1* as “pathogenic” or “benign” (Choufani et al. 2015). To date, many publications have shown that DNA methylation signatures provide a unique means of assessing VUS in numerous syndromes. This utility is especially germane given that the expanded use of DNA-sequence-based diagnostics has resulted in a substantial increase in VUS identification. Most diagnostic laboratories use the American College of Medical Genetics (ACMG) guidelines for VUS interpretation, which include in silico prediction tools with sensitivity and specificity as low as 33% (Niroula and Vihinen 2019). While there are currently no gold standards for generation of DNA methylation signatures or clinically acceptable thresholds of sensitivity and specificity for variant interpretation, our studies and others have shown that the implementation of DNA methylation signatures into diagnostics can address the issue of variant interpretation for potentially hundreds of gene variants associated with rare neurodevelopmental syndromes.

**Fig. 1:**
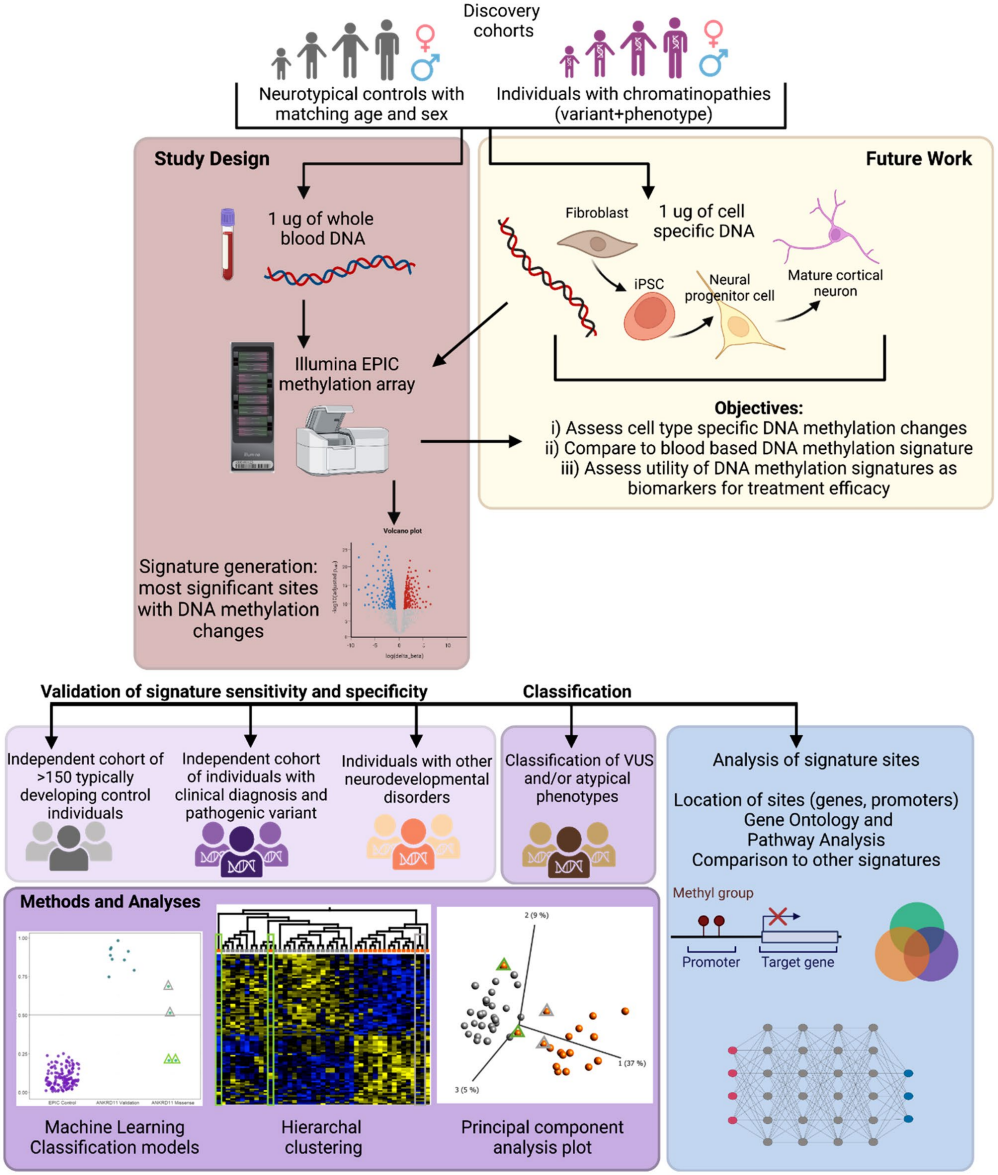
Generating a new DNA methylation signature for a specific chromatinopathy, begins with obtaining DNA samples from individuals clinically diagnosed with that specific chromatinopathy and control individuals. The resulting DNA methylation signature is then validated in an independent cohort of individuals with the same chromatinopathy, which provides a sensitivity estimate. Specificity of the signature is then estimated by analyzing methylation at signature sites in a large independent cohort of controls and cohorts of individuals diagnosed with other chromatinopathies. In the bottom plot, the KBG syndrome signature demonstrates high sensitivity and specificity, as the KBG validation cohort are classified as “case-like” with high prediction scores, whereas control individuals (validation set) and individuals with other diagnoses, Kabuki and Weaver syndromes, are classified as “control-like” with low prediction scores. As with all signatures, a classification of “control-like” means an individual does not have a casual variant for that specific chromatinopathy; it does not mean that the individual has no diagnosable chromatinopathies. To determine an individual’s epigenotype at signature sites, we utilize a combination of principal component analysis, hierarchal clustering, and machine learning models. Once a DNA methylation signature is generated for a chromatinopathy, it is annotated to identify the location of sites in the genome including overlapping genes and specific location within gene structure (promoter, TSS, body, UTR). Pathway enrichment and gene ontology can elucidate which biological processes and molecular functions genes underlying signature sites are involved in. Comparison to other DNA methylation signatures for chromatinopathies can potentially identify common methylation changes and gene targets relevant to exploring therapeutics in the future

To investigate an individual’s DNA methylation profile or “epigenotype” on a syndrome-specific DNA methylation signature, different analytical tools can be useful. A commonly used approach for variant classification uses machine learning models, wherein a classification model is trained on DNA methylation profiles of the reference or discovery cohorts (cases and controls) at established signature sites. The so-called classifier generates a probability score between 0 and 100% for each variant tested; this score represents the likelihood that a variant is pathogenic and therefore “matches” DNA methylation profiles from individuals with definitive molecular and clinical diagnoses of the neurodevelopmental syndrome in question. For interpretation, these probabilities are binned into three categories: high probabilities are deemed “pathogenic” with “disease-like” DNA methylation profiles, low probabilities are deemed “benign” with “control-like” DNA methylation profiles, and medium probabilities are deemed “intermediate” and require further investigation. In addition to machine learning models, principal component analysis (PCA) and hierarchical clustering, are tools that provide additional information to more accurately characterize an individual’s epigenotype, particularly for those with intermediate classification scores (Fig. 1). Both PCA and hierarchical clustering are helpful tools to visualize patterns based on DNA methylation profiles at specific signature sites. As these statistical tools are not easily employed by clinicians and researchers, many have been packaged into user-friendly platforms. In our research group, we have generated DNA methylation signatures for more than 40 neurodevelopmental syndromes associated with pathogenic variants in epigenetic regulatory genes and cataloged many in EpigenCentral (Turinsky et al. 2020). This open access web-based platform was designed to accelerate epigenetics research, specifically variant classification in neurodevelopmental syndromes (Turinsky et al. 2020). In the sections below, we describe examples of various types of classifications and the knowledge that can be gained from these results, with respect to complex genotype–phenotype relationships.

## Not all benign classification scores indicate a benign variant

While an individual’s classification score might indicate a “benign” variant via machine learning on a given signature, further assessment of the DNA methylation profile at signature sites using other tools can identify unique subgroups or patterns of DNA methylation with biological relevance to pathophysiology. This emphasizes the importance of using a variety of analytic tools for classifying DNA methylation profiles. Below, we describe two studies for which further investigation of a “benign” classification elucidated the pathogenicity of a sequence variant.

In the first study, we identified an individual with an undergrowth phenotype and a VUS [p.Ala738Thr] in *EZH2* [MIM: 601573]*,* a gene in which pathogenic variants are associated with Weaver syndrome [MIM: 277590], characterized by an overgrowth phenotype. This individual’s classification score using the Weaver syndrome signature was control-like (Choufani et al. 2020). Further assessment using hierarchical clustering at Weaver syndrome signature sites revealed this individual had the opposite methylation pattern to that of individuals with Weaver syndrome and is also different from controls (Choufani et al. 2020). We hypothesized the VUS had a gain-of-function effect on EZH2, opposite to the loss-of function effect of variants causing Weaver syndrome. We measured the enzymatic activity of EZH2 using an in vitro luminescence assay in control cells and cells carrying the VUS [p.Ala738Thr] in *EZH2* and demonstrated that the cells with the VUS had significantly higher enzymatic activity than the control cells (Choufani et al. 2020). The results of this functional assay supported our hypothesis and were congruent with the clinical phenotype and DNA methylation profile which were opposite to those observed in individuals with Weaver syndrome.

In another study, we established a DNA methylation signature specific to individuals with Floating Harbor syndrome [FLHS; MIM:136140] caused by pathogenic variants in exons 33/34 of the *SRCAP* [MIM:611421] gene (Rots et al. 2021). The DNA methylation profiles of nine individuals with pathogenic variants proximal to exon 33 had control-like classification scores. However, these proximal variants clearly clustered into a separate subgroup using PCA and hierarchical clustering. That is, they were distinguishable from both controls and individuals with FLHS (Rots et al. 2021). In addition to this unique clustering pattern, variants proximal to exon 33 were also associated with a common clinical presentation that was distinct from FLHS. We ultimately described a novel neurodevelopmental syndrome [MIM: 619595] associated with a unique DNA methylation signature (Rots et al. 2021). To that end, for variants with control-like classification scores, it is also critical to review the individual’s molecular and clinical data, and to assess differences and similarities with respect to the neurodevelopmental syndrome in question.

The combination of multiple analytical tools (PCA, hierarchical clustering, classification models) to investigate DNA methylation differences is important for a robust interpretation of one’s “epigenotype”. This is necessary to ascertain whether the variant is truly benign or if the variant impacts the protein via a different pathogenic mechanism likely associated with a unique DNA methylation pattern and a distinguishable clinical phenotype. Functional assays in vitro can be a challenging research investment but are ultimately required to understand the effect of different variants on the protein. As an adjunct to genome sequencing technologies and in vitro functional assays, DNA methylation signatures have proven utility as a functional assay that can be used to support variant classification.

## Variant interpretation using DNA methylation signatures is not binary

In early studies of DNA methylation signatures, profiles of test cases often exhibited very high classification scores (disease-like; > 70%) or very low classification scores (control-like; < 30%), so-called “intermediate” scores were described later. This binary test output reflected, in part, researchers’ and consulting clinicians’ expectations of a yes or no outcome, indicating whether or not a given variant is or is not the causal variant for the observed phenotype. However, with the rapid development of signatures and variant classification, variants with intermediate classification scores (35–65%) became more prevalent, highlighting DNA methylation profiles that did not resemble the profiles of either reference cases or controls, with high confidence.

In a series of studies, we found that intermediate classifications often reflected important biologic and phenotypic differences. Our research group first identified an intermediate score for an individual with a VUS in *SMARCA2* [MIM:600014]*.* This individual’s variant was distal to the ATPase/Helicase domain, in which most pathogenic variants causing Nicolaides-Baraitser syndrome [MIM:601358] occur (Chater-Diehl et al. 2019). We determined that this individual’s DNA methylation profile was intermediate due to a subset of signature sites showing methylation levels matching that of typically developing individuals and other sites matching levels of individuals with Nicolaides-Baraitser syndrome, a pattern that became apparent using hierarchical clustering (Chater-Diehl et al. 2019). DNA methylation findings were congruent with the reported phenotype for this individual, in that they exhibited a milder neurodevelopmental phenotype. From this study, we concluded that variable phenotypic expressivity can be reflected in DNA methylation profiling (i.e., milder phenotype-intermediate score) and can potentially be attributed to the variant location and/or type. We encountered similar findings in methylation analysis of Au-Kline syndrome [MIM: 616580] typically caused by pathogenic truncating variants in the *HNRNPK* [MIM:600712] gene. We identified individuals (*n* = 11) with de novo missense *HNRNPK* variants and mild phenotypes to have intermediate classification scores (34–52%) (Choufani et al. 2022). Individuals with intermediate classification scores were found to have DNA methylation changes in the same direction as the test cases with high classification scores but of smaller magnitude, as evidenced by follow-up analysis using hierarchical clustering and PCA (Choufani et al. 2022). These findings support the hypothesis, variant type and/or location and associated milder phenotypes can be correlated with intermediate DNA methylation classification scores.

Intermediate classification scores can also reflect somatic mosaicism. Using the Weaver syndrome DNA methylation signature, we classified the DNA methylation profiles of a father–child pair with a missense variant in *EZH2*, and found discordant classification scores of 49% and 82%, respectively (Choufani et al. 2020). Across all signature sites, the father had a smaller magnitude of DNA methylation change compared to his child. Phenotypically, the child had a clinical diagnosis of Weaver syndrome, whereas the father presented with tall stature but no other features of Weaver syndrome. We examined the duo’s blood DNA for somatic mosaicism and found the percentage of the variant allele to be 46% in the child and 38% in the father (Choufani et al. 2020). While variable phenotypic expressivity of inherited variants can be attributed to somatic mosaicism in some instances, in others intrafamilial phenotypic heterogeneity may be a characteristic of the causal genetic variants associated with that syndrome (Schirwani et al. 2022; van der Spek et al. 2022). Intrafamilial phenotypic heterogeneity has been reported in KBG syndrome [MIM:148050], and in our recent study, we identified intermediate classification scores for a mother–child pair, where the mother was considered unaffected (51%) and the child was considered mildly affected (68%) (Awamleh et al. 2022a) (Fig. 1). Somatic mosaicism was not detected in the mother’s blood; however, this does not exclude the possibility of mosaicism in the mother in tissues other than blood. These studies highlight the multitude of biological phenomena that may result in “intermediate” classifications of inherited and de novo variants, including mosaicism, intrafamilial phenotypic heterogeneity, and likely hypomorphic variants in syndromes typically caused by haploinsufficiency.

## Further complexities: signature specificity and functionally related genes

The idea that DNA methylation signatures are unique to each syndrome is not entirely accurate. We first observed this when studying variants in the genes for Kabuki syndrome 1 [MIM: 147920] and Kabuki syndrome 2 [MIM: 300867], *KMT2D* [MIM:602113] and *KDM6A* [MIM: 300128], respectively. We developed a DNA methylation signature for Kabuki syndrome 1 and found that a *KDM6A* variant causing Kabuki syndrome 2 had a disease-like classification score at Kabuki syndrome 1 signature sites (Butcher et al. 2017). *KDM6A* and *KMT2D* encode different histone modifier proteins, that function as a part of a multi-protein complex. That is, the two genes are known to co-regulate a common set of target genes (Kim et al. 2014). Based on these findings we hypothesized that pathogenic variants in functionally related genes have partially overlapping signatures.

We encountered this phenomenon again using the DNA methylation signature for Weaver syndrome, caused by pathogenic variants in *EZH2*, where variants in *SUZ12* and *EED* had high classification scores (> 90%) (Choufani et al. 2020). All three genes are core members of the polycomb repressive complex 2, responsible for methylation of histone 3 lysine 27. Similarly at the DNA methylation signature for Bohring-Opitz syndrome [BOS; MIM: 605039] caused by pathogenic variants in *ASXL1* [MIM: 612990]*,* an individual with Shashi-Pena syndrome [MIM: 617190] and a variant in *ASXL2* [MIM: 617190] also had a high classification score (84%) (Awamleh et al. 2022b). Both genes belong to the same gene family and encode proteins that function in the polycomb repressive deubiquitinase complex, responsible for the deubiquitination of histone 2A at lysine 119. Once sufficient case numbers are available, we can attempt to establish syndrome-specific signatures and assess the degree to which the signatures overlap. Until then, DNA methylation signatures of functionally relevant genes can potentially be used to assess variant pathogenicity. Ongoing efforts to establish relationships between DNA methylation signatures and singular genes or subregions of genes or to specific phenotypes, will inform current complexities in syndrome terminology, i.e. naming syndromes to appropriately reflect molecular etiology and phenotype. In future, DNA methylation patterns will provide another layer of information can be used when considering if a ‘Mendelian disorder entity’ is distinct or if it overlaps with a previously described condition (Biesecker et al. 2021).

## Expectations for the future of DNA methylation signatures

To date, DNA methylation signatures have shown tremendous promise as a diagnostic tool, however, it is important to consider their limitations. The strength of the DNA methylation signal associated with neurodevelopmental syndromes can be easily overcome by other biological factors strongly associated with DNA methylation, such as age at tissue collection and cell type. We have demonstrated that substandard experimental design can generate a DNA methylation signature that misclassifies variants, and that robust experimental design is essential for identifying DNA methylation signatures that exhibit both stability and utility (Chater-Diehl et al. 2021). We previously published a framework for DNA methylation signature development that includes considerations on sample size and statistical parameters. We emphasized the importance of considering the complex relationships between genetic variants and phenotypes for each syndrome, which can be optimized via collaborations between clinicians and researchers (Chater-Diehl et al. 2021).

We expect that there is more to learn about the complex relationships between genotype, phenotype and epigenotype. For example, future analysis of DNA methylation profiles in neurodevelopmental syndromes could be used to further stratify individuals based on the presence/absence of a sub-phenotype or the severity of a particular phenotype, which would be relevant for predicting outcomes and exploring potential therapeutic interventions. The utility of DNA methylation signatures as biomarkers for treatment or to identify potential treatment targets is an area that has not yet been sufficiently investigated. The assessment of treatment efficacy can be carried out in cell and/or animal models. Profiling DNA methylation in biologically relevant cell models such as neural precursor cells or mature cortical neurons will be a critical first step to identify whether blood-based signatures are translatable to other cell types and if there are cell-type-specific DNA methylation signatures (Fig. 1). In addition, mouse models of neurodevelopmental disorders are another research approach that can answer questions about tissue/cell specificity of DNA methylation signatures and the potential utility of signatures as biomarkers of treatment efficacy. Lastly, advancements in genome sequencing will likely improve the efficacy of genetic diagnostics in the next decade. Future developments in the use of long read sequencing could authenticate platforms that simultaneously capture both genomic and DNA methylation changes for clinical grade genetic testing.

Collectively, the data presented here demonstrate how DNA methylation signatures are a powerful functional tool for variant interpretation in neurodevelopmental syndromes and for the delineation of complex genotype–phenotype relationships. In addition to signature development in blood for variant classification, future studies should focus on assessing how blood-based signatures are translatable to other cell and tissue types, and whether they have additional applications to potentially identify therapeutic targets or act as early treatment biomarkers for critical outcome measures.
